# The Pan-Genome of the Animal Pathogen *Corynebacterium pseudotuberculosis* Reveals Differences in Genome Plasticity between the Biovar *ovis* and *equi* Strains

**DOI:** 10.1371/journal.pone.0053818

**Published:** 2013-01-14

**Authors:** Siomar C. Soares, Artur Silva, Eva Trost, Jochen Blom, Rommel Ramos, Adriana Carneiro, Amjad Ali, Anderson R. Santos, Anne C. Pinto, Carlos Diniz, Eudes G. V. Barbosa, Fernanda A. Dorella, Flávia Aburjaile, Flávia S. Rocha, Karina K. F. Nascimento, Luís C. Guimarães, Sintia Almeida, Syed S. Hassan, Syeda M. Bakhtiar, Ulisses P. Pereira, Vinicius A. C. Abreu, Maria P. C. Schneider, Anderson Miyoshi, Andreas Tauch, Vasco Azevedo

**Affiliations:** 1 Department of General Biology, Federal University of Minas Gerais, Belo Horizonte, Minas Gerais, Brazil; 2 Center for Biotechnology, Bielefeld University, Bielefeld, Nordrhein-Westfalen, Germany; 3 CLIB Graduate Cluster Industrial Biotechnology, Center for Biotechnology, Bielefeld University, Bielefeld, Nordrhein-Westfalen, Germany; 4 Department of Genetics, Federal University of Pará, Belém, Pará, Brazil; 5 Department of Veterinary Medicine, Federal University of Lavras, Lavras, Brazil; St. Petersburg Pasteur Institute, Russian Federation

## Abstract

*Corynebacterium pseudotuberculosis* is a facultative intracellular pathogen and the causative agent of several infectious and contagious chronic diseases, including caseous lymphadenitis, ulcerative lymphangitis, mastitis, and edematous skin disease, in a broad spectrum of hosts. In addition, *Corynebacterium pseudotuberculosis* infections pose a rising worldwide economic problem in ruminants. The complete genome sequences of 15 *C. pseudotuberculosis* strains isolated from different hosts and countries were comparatively analyzed using a pan-genomic strategy. Phylogenomic, pan-genomic, core genomic, and singleton analyses revealed close relationships among pathogenic corynebacteria, the clonal-like behavior of *C. pseudotuberculosis* and slow increases in the sizes of pan-genomes. According to extrapolations based on the pan-genomes, core genomes and singletons, the *C. pseudotuberculosis* biovar *ovis* shows a more clonal-like behavior than the *C. pseudotuberculosis* biovar *equi*. Most of the variable genes of the biovar *ovis* strains were acquired in a block through horizontal gene transfer and are highly conserved, whereas the biovar *equi* strains contain great variability, both intra- and inter-biovar, in the 16 detected pathogenicity islands (PAIs). With respect to the gene content of the PAIs, the most interesting finding is the high similarity of the pilus genes in the biovar *ovis* strains compared with the great variability of these genes in the biovar *equi* strains. Concluding, the polymerization of complete pilus structures in biovar *ovis* could be responsible for a remarkable ability of these strains to spread throughout host tissues and penetrate cells to live intracellularly, in contrast with the biovar *equi*, which rarely attacks visceral organs. Intracellularly, the biovar *ovis* strains are expected to have less contact with other organisms than the biovar *equi* strains, thereby explaining the significant clonal-like behavior of the biovar *ovis* strains.

## Introduction

The genus *Corynebacterium* belongs to the CMNR group from the supra-generic group of *Actinomycetes*, which includes genera of great medical, veterinary, and biotechnological importance, such as *Corynebacterium*, *Mycobacterium*, *Nocardia*, and *Rhodococcus*. These genera have specific features in common, such as a high DNA G+C content and a specific organization of the cell wall, which is mainly composed of peptidoglycans, arabinogalactans, and mycolic acids [1]. The genus *Corynebacterium* was originally created to include *Corynebacterium diphtheriae* and other pathogenic species [2]. Several other bacteria that differed in shape, pathogenicity and sporulation were later added to this group [3]. Currently, the genus is composed of pathogenic species such as *Corynebacterium diphtheriae*, the causative agent of diphtheria [4]; opportunistic pathogens such as *Corynebacterium jeikeium*, which is responsible for some nosocomial infections in humans [5]; and non-pathogenic species such as *Corynebacterium glutamicum*, which is highly utilized in industrial amino acid production [6].

*Corynebacterium pseudotuberculosis* is a facultative intracellular and pleomorphic member of the genus *Corynebacterium*. This bacterium is non-motile, although it does possess fimbriae, and it is the causative agent of caseous lymphadenitis (CLA) in sheep and goats [7]. A close taxonomic relationship between *C. pseudotuberculosis* and *Corynebacterium ulcerans* has been suggested because these organisms are the only corynebacteria that produce the exotoxin phospholipase D [8], [9]. Moreover, some strains of *C. pseudotuberculosis* and *C. ulcerans* express the diphtheria toxin, which indicates a relationship between both species and *C. diphtheriae* [10]. This relationship has also been demonstrated by a phylogenetic analysis of the *rpoB* gene [1]. The initial classification of *C. pseudotuberculosis* was based on morphological and biochemical characteristics [7], [11]: the results of the nitrate reduction test play an important role in distinguishing the biovar *ovis* (isolated from sheep and goats; negative nitrate reduction) from the biovar *equi* (isolated from horses and bovines; positive nitrate reduction) [12].

In sheep and goats, *C. pseudotuberculosis* biovar *ovis* strains are responsible for causing the aforementioned infectious, contagious, chronic disease CLA, which is mainly characterized by the presence of caseous necrosis on the lymphatic glands or abscess formation in superficial lymph nodes and subcutaneous tissues [13]. CLA is a widespread disease that has been reported in several countries, including Australia, Brazil, Canada, New Zealand, South Africa, and the United States, where sheep and goat farming are prevalent [1], [14]–[18]. CLA produces economic losses for sheep and goat farmers by causing skin deterioration and reducing yields of milk and wool. In addition to these effects, the visceral form of the disease can affect internal organs, resulting in weight loss, carcass condemnation and death [19]. The disease is transmitted through direct contact with superficial wounds, which can be the result of common procedures such as castration and shearing [20]. The transmission and dissemination of *C. pseudotuberculosis* are also associated with the following: a high resistance to environmental conditions [21]–[23]; a low detection rate, with the visceral form of the disease usually being detected in the later stages or in the slaughterhouse [24]; the inefficacy of antibiotic therapies due to abscess formation and an intra-macrophagic lifestyle [25]; high variability in the severity of the disease in vaccinated animals and in the protection levels of the vaccines [26]; and the variable efficacy of licensed vaccines, which are intended for use in sheep, in goat immunizations [27].

Although *C. pseudotuberculosis* was initially identified as causing CLA in sheep and goats, this bacterium has also been isolated from other species that exhibit different symptoms, including horses, cows, camels, buffalo, and even humans [1], [28]–[30]. Despite the broad host spectrum, natural cross-species transmission of *C. pseudotuberculosis* between small ruminants and cattle does not appear to occur [12], although infections of cattle with both biovars have been previously reported [31].

*C. pseudotuberculosis* infections in horses can display three different disease patterns: external abscesses (pigeon fever), ulcerative lymphangitis of the limbs, and a visceral form that affects the internal organs [32], [33]. Additionally, several clinical symptons of the diseases caused by *C. pseudotuberculosis* have been described in cattle: pyogranulomatous reactions, abscess formation, mastitis, visceral commitment, and necrotic and ulcerative dermatitis on the heel of the foot, which is accompanied by edematous swelling and lameness [24]. In bulls and buffalo, there is evidence of the mechanical transmission of *C. pseudotuberculosis* by houseﬂies or other diptera, in addition to transmission via skin contact between animals [23], [24], [34]–[37]. Moreover, all reported outbreaks of CLA in horses in the United States have been preceded by large populations of houseﬂies and other diptera during the summer, a phenomenon promoted by high environmental temperatures and drought conditions [38] that may also be related to a rise in the number of affected herds in Israel [24].

Although the pathogenic mechanism of CLA is well understood, there remains a lack of information about the virulence factors of *C. pseudotuberculosis* and the pathogenic mechanisms of the other diseases caused by this bacterium [1], [39], [40]. Virulence factors play an important role in the adhesion, invasion, colonization, spread inside the host, and immune system evasion of pathogenic bacteria; they also allow contact, penetration and survival inside the host [41]. Billington *et al.* [42] reported four *C. pseudotuberculosis* genetic factors, the *fagABC* operon and the *fagD* gene, that play an important role in virulence; they are involved in iron acquirement and, therefore, enable the bacterium to survive in environments where iron is scarce. The *fagABC* operon and the *fagD* gene are found in a pathogenicity island along with the *pld* gene, which encodes phospholipase D (PLD) [43]. PLD is the primary virulence factor of *C. pseudotuberculosis*; it promotes the hydrolysis and degradation of sphingomyelin in endothelial cell membranes, which increases vascular permeability and contributes to the spread and persistence of the bacterium in the host [27], [44], [45]. More recently, Trost *et al.* [46] reported the presence of two pilus gene clusters in the *C. pseudotuberculosis* FRC41 strain, which is in agreement with the previously reported visualization of pilin structures in other strains of *C. pseudotuberculosis* [47]. Pili are helical, cylinder-shaped structures, which are observed attached to and protruding from the bacterial cell surface. Pili play an important role in virulence as they enable pathogens to bind to molecules on various host tissues. After attaching to the host cell surface, the pathogen is able to initiate specific biochemical processes, such as extracellular and intracellular invasion, that will result in its proliferation in and dissemination among the host tissues [48].

To better understand the different symptoms of *C. pseudotuberculosis* infections in the broad spectrum of hosts and how genome plasticity is related to the symptom patterns, we performed pan-genomic comparative analyses of 15 *C. pseudotuberculosis* strains. In the following sections, we present the phylogenomic correlations between *C. pseudotuberculosis* and other corynebacteria. Furthermore, we describe the content and extrapolations of the following gene subsets from *C. pseudotuberculosis*: the “pan-genome”, which is the complete inventory of genes found in any member of the species; the “core genome”, which is composed of the genes that are present in all the species strains and that are thus important for basic life processes; and the “singletons”, which represent genes found only in a given strain. Finally, we provide insights into the specific subsets (singletons and the pan- and core genomes) of both biovars of *C. pseudotuberculosis*, *ovis* and *equi*, and we correlate these subsets with the plasticity of pathogenicity islands, virulence genes, and biovar-specific diseases.

## Materials and Methods

### Genome Sequences

The genome sequences of 15 *C. pseudotuberculosis* strains were retrieved from the NCBI database (http://www.ncbi.nlm.nih.gov/genbank/): 9 biovar *ovis* strains, which were isolated from sheep, goats, humans, llamas, antelopes, and cows, and 6 biovar *equi* strains, which were isolated from horses, camels, and buffalo (Table 1). The strains were isolated in Oceania (Australia), South America (Brazil and Argentina), North America (United States), Africa (South Africa, Egypt and Kenya), southwestern Asia (Israel), and Europe (the United Kingdom, Belgium, France and Scotland). The clinical symptoms of infections with these strains vary broadly and include abscesses, mastitis, lymphangitis, necrogranuloma, and edematous skin disease (Table 1).

**Table 1:** General information about the 15 *C. pseudotuberculosis* strains used in this work.

Strains	Biovar	Host	Countryof isolation	Clinicaldescription	Genomesize	Numberof genes	Singletons	GenBankaccessionN°	Reference
1002	*ovis*	Goat	Brazil	CLA abscess	2,335,113	2,203	0	CP001809	[43]
C231	*ovis*	Sheep	Australia	CLA abscess	2,328,208	2,204	3	CP001829	[43]
42/02-A	*ovis*	Sheep	Australia	CLA abscess	2,337,606	2,164	5	CP003062	[49]
PAT10	*ovis*	Sheep	Argentina	Lung abscess	2,335,323	2,200	1	CP002924	[50]
3/99-5	*ovis*	Sheep	Scotland	CLA	2,337,938	2,239	39	CP003152	[49]
267	*ovis*	Llama	USA	CLA abscess	2,337,628	2,249	8	CP003407	[51]
P54B96	*ovis*	Antelope	South Africa	CLA abscess	2,337,657	2,205	2	CP003385	–
I19	*ovis*	Cow	Israel	Bovine mastitis abscess	2,337,730	2,213	0	CP002251	[52]
FRC41	*ovis*	Human	France	Necrotizing lymphadenitis	2,337,913	2,171	12	CP002097	[46]
CIP52.97	*equi*	Horse	Kenya	Ulcerative lymphangitis	2,320,595	2,194	30	CP003061	[53]
316	*equi*	Horse	USA	Abscess	2,310,415	2,234	25	CP003077	[54], [55]
258	*equi*	Horse	Belgium	Ulcerative lymphangitis	2,314,404	2,195	29	CP003540	[56]
1/06-A	*equi*	Horse	USA	Abscess	2,279,118	2,127	20	CP003082	[57]
Cp162	*equi*	Camel	UK	Neck abscess	2,293,464	2,150	13	CP003652	[58]
31	*equi*	Buffalo	Egypt	Abscess	2,297,010	2,170	50	CP003421	[59]

### *Corynebacterium* Genus Phylogenomic Analyses

The Gegenees (version 1.1.4) software was used to perform the phylogenomic analyses at the genus level and to retrieve the GenBank sequences of all the complete *Corynebacterium* genomes from the NCBI ftp site. Briefly, Gegenees was used to divide the genomes into small sequences and to perform an all-versus-all similarity search to determine the minimum content shared by all the genomes. Next, the minimum shared content was subtracted from all the genomes, resulting in the variable content, which was compared with all the other strains to generate the percentages of similarity. Finally, these percentages were plotted in a heatmap chart with a spectrum ranging from red (low similarity) to green (high similarity) [60]. The Gegenees data can also be exported as a distance matrix file in nexus format. Here, we used the distance matrix as an input file for the SplitsTree (version 4.12.6) software to generate a phylogenomic tree using the UPGMA method [61], [62].

### Pan-genome, Core Genome and Singleton Analyses

This section describes the analyses that were performed for all of the following three datasets: A) all strains, using *C. pseudotuberculosis* strain 1002 as a reference; B) the biovar *ovis* strains, using *C. pseudotuberculosis* strain 1002 as a reference; and C) the biovar *equi* strains, using *C. pseudotuberculosis* strain CIP52.97 as a reference. To calculate the pan-genome, core genome and singletons of the *C. pseudotuberculosis* species, we used EDGAR (version 1.2), multiple-strain genome comparison software that performs homology analyses based on a specific cutoff that is automatically adjusted to the query dataset [63]. Initially, the genome sequences of *C. pseudotuberculosis* were retrieved from GenBank, and a new project was created on the annotation platform GenDB (version 2.4) to homogenize the genome annotations [64]. Subsequently, an EDGAR project was created based on the GenDB annotations, and homology calculations based on BLAST Score Ratio Values (SRVs) were performed. According to the SRV method, instead of using raw BLAST scores or E-values, a normalization of each BLAST bit score is calculated by considering the maximum possible bit score (i.e., the bit score of the subject gene against itself). This results in a value ranging from 0 to 1 [65], which is multiplied by 100 and rounded in a percentage value of homology. Finally, a sliding window on the SRV distribution pattern was used to automatically calculate the SRV cutoff with EDGAR [63]. For this work, a SRV cuttof of 59 was estimated. Pairs of genes exhibiting a Bidirectional Best Hit where both single hits have a SRV higher than the specific cutoff were considered to be orthologous genes.

The core genome was calculated as the subset of genes presenting orthologs in all the selected strains. The gene set of subject strain A was compared with the gene set of query strain B, and only genes with orthologs in both strains were members of core AB. The resulting subset was then compared with the gene set of query strain C, and the comparisons continued in a reductive manner. The pan-genome was calculated in the same way, but in an additive manner: the initial pan-genome was composed of strain A, and the non-orthologous genes of strain B were added to pan-genome A to create the pan-genome AB. The resulting set of genes was then compared with strain C, and the comparisons continued in the same manner. Finally, the singletons were calculated as genes that were present in only one strain and thus did not present orthologs in any other *C. pseudotuberculosis* sequenced strain.

The developments of the core genome, pan-genome and singletons of *C. pseudotuberculosis* were calculated based on permutations of all the sequenced genomes. The developments of the core genome and singletons were calculated using the least-squares fit of the exponential regression decay to the mean values. In contrast, the statistical computing language R was used to calculate the pan-genome extrapolation using Heaps’ Law by estimating the parameters κ and γ using the nonlinear least-squares curve fit to the mean values [66], [67].

The core genes of all the strains, including the biovar *ovis* strains and the biovar *equi* strains, were classified by their Cluster of Orthologous Genes (COG) functional category as the following: 1. information storage and processing; 2. cellular processes and signaling; 3. metabolism; and 4. poorly characterized. To perform this analysis, the query sets of core genes were submitted to BLAST protein (blastp) similarity searches against the COG database, the proteins with E-values higher than 10^−6^ were discarded, and the best BLAST results for each protein were considered for the COG functional category information retrieval. Finally, the whole-genome comparison maps were visualized using the software CGView Comparison Tool (CCT) [68]. All the strains were plotted against *C. pseudotuberculosis* strains 1002 and CIP52.97 to generate two genome comparison maps.

### Pathogenicity Island Prediction

The plasticity of the 15 genomes was assessed using PIPS: Pathogenicity Island Prediction Software (version 1.1.2). PIPS is a multi-pronged approach that predicts pathogenicity islands (PAIs) based on common features, such as G+C content, codon usage deviation, high concentrations of virulence factors and hypothetical proteins, the presence of transposases and tRNA flanking sequences, and the absence of the query region in non-pathogenic organisms of the same genus or related species [69]. *C. glutamicum* strain ATCC 13032 was selected as the non-pathogenic organism of the same genus [6], and separate predictions were performed for each strain. The sizes of the islands were compared with those of all the other strains via ACT: Artemis Comparison Tool (version 10.2.0) and CCT [68], [70]. Following the curation of the PAIs, the genes of all the islands in each strain were assessed for their presence/absence in all the other strains using the pan-genome data generated by EDGAR. The overall number of genes in the PAIs of the subject strain that were shared by the query strains was expressed as a percentage and plotted in a heatmap. The percentages were also converted into a nexus file, which was used in SplitsTree (version 4.12.6) to create a phylogenomic tree using the UPGMA method [61], [62]. Finally, zoomed PAI figures were created using a script from CCT (create_zoomed_maps.sh) with the zoom option selected as 30×.

## Results

### Phylogenomics of the Genus *Corynebacterium* and *C. pseudotuberculosis* Biovars

**Figure 1:**
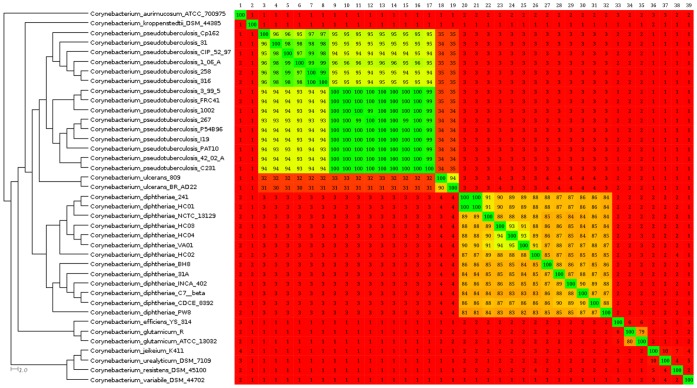
Phylogenomic tree and heatmap analyses of the genus *Corynebacterium*. All the complete genomes from the genus *Corynebacterium* were retrieved from the NCBI ftp site. Comparisons between the variable content of all the strains were plotted as percentages of similarity on the heatmap using Gegenees (version 1.1.4). The percentage of similarity was used to generate a phylogenomic tree with SplitsTree (version 4.12.6). Numbers from 1 to 39 (upper-left to upper-right corner) represent species from *Corynebacterium aurimucosum* ATCC 70097 to *Corynebacterium variable* DSM 44702 (upper-left to lower-left corner). Percentages were plotted with a spectrum ranging from red (low similarity) to green (high similarity). On the heatmap, the upper portion is not symmetrical to the lower portion because the variable contents of all genomes present different sizes. Therefore, considering a scenario where the variable content from genomes A and B are composed of 100 and 80 genes, respectively, with a common repertoire of 40 genes, genome A will present 40% of similarity to genome B and genome B will present 50% of similarity to genome A.

To evaluate the phylogenomic relationships between *C. pseudotuberculosis* strains and other species of the genus *Corynebacterium*, the *Corynebacterium* shared gene content was automatically determined using Gegenees. Then, the shared gene content was subtracted from all genomes and the resulting variable content of each genome sequence was cross-compared to generate a phylogenomic tree and to plot a heatmap (Figure 1). According to the generated phylogenomic tree, the pathogenic species *C. diphtheriae*, *C. pseudotuberculosis*, and *C. ulcerans* formed three closely related clusters. Moreover, *C. glutamicum* and *Corynebacterium efficiens*, two non-pathogenic bacteria of great industrial importance as amino acid producers [6], [71], appeared closely related in a different cluster. Additionally, *Corynebacterium kroppenstedtii*, another pathogenic bacterium of the *Corynebacterium* genus, was positioned between the clusters of pathogens (*C. pseudotuberculosis*, *C. diphtheriae* and *C. ulcerans*) and non-pathogens (*C. glutamicum* and *C. efficiens*). Finally, the opportunistic bacteria *C. jeikeium*, *Corynebacterium urealyticum* and *Corynebacterium resistens* [5], [72], [73] clustered together with the non-pathogenic *Corynebacterium variabile* [74], whereas *Corynebacterium aurimucosum* formed a new branch [75].

At the species level, the *C. pseudotuberculosis* genomes clustered in two separate groups representing the two biovars of the species: biovar *ovis*, with more than 99% similarity according to the heatmap; and biovar *equi*, with a similarity ranging from 95% to 100%. Moreover, the heatmap indicated an almost clonal-like behavior of *C. pseudotuberculosis* compared with the *C. diphtheriae* species, which presented similarities raging from 82% to 100%.

**Figure 2:**
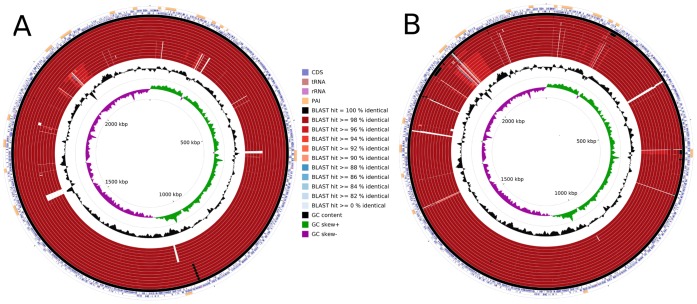
Comparative genomic maps of the *C. pseudotuberculosis* biovar *equi* and *ovis* strains. A, all the *C. pseudotuberculosis* strains were aligned using *C. pseudotuberculosis* strain 1002 as a reference. From the inner to outer circle on A: the biovar *equi* strains Cp31, Cp1/06-A, CpCp162, Cp258, Cp316 and CpCIP52.97; and, the biovar *ovis* strains CpC231, CpP54B96, Cp267, CpPAT10, CpI19, Cp42/02-A, Cp3/99-5, CpFRC41 and Cp1002. B, all the *C. pseudotuberculosis* strains were aligned using *C. pseudotuberculosis* strain CIP52.97 as a reference. From the inner to outer circle on B: the biovar *ovis* strains CpC231, Cp1002, CpPAT10, Cp267, CpP54B96, CpI19, Cp42/02-A, CpFRC41, Cp3/99-5; and, the biovar *equi* strains Cp1/06-A Cp31, CpCp162, Cp316, Cp258 and CpCIP52.97. CDS, coding sequences; tRNA, transfer RNA; rRNA, ribosomal RNA; and PAI, pathogenicity island.

An alternative to assess the clonal-like behavior of species is the use of a circular genome comparison, which was performed with the software CCT. The results reveal regions of plasticity based on a chosen reference and, interestingly, plot the genomes from outer to inner circles by order of decreasing similarity. As shown in Figure 2, we plotted all the genomes using *C. pseudotuberculosis* strain 1002 (bv. *ovis*) and *C. pseudotuberculosis* strain CIP52.97 (bv. *equi*) as references. Figure 2A shows specific patterns of deletions in all the biovar *equi* strains compared with *C. pseudotuberculosis* 1002. In Figure 2B, however, the deletions in the comparison with *C. pseudotuberculosis* CIP52.97 are not specific to particular biovars, but rather are generalized. In both cases, the genomes that were classified as having the same biovar as the reference strain were clustered together in the outer circles, whereas the other strains were clustered in the inner circles.

### The Pan-genome of the Species *C. pseudotuberculosis*

**Figure 3:**
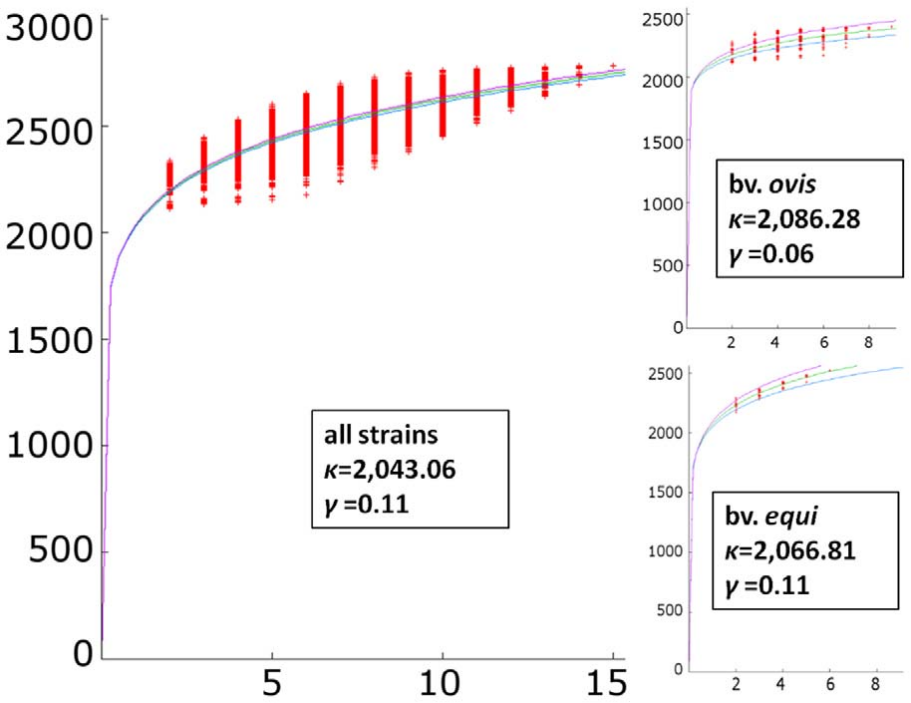
Pan-genome development of *C. pseudotuberculosis*. Center chart, the pan-genome development using permutations of all 15 strains of *C. pseudotuberculosis*; upper-right chart, the pan-genome development of the *C. pseudotuberculosis* biovar *ovis* strains; lower-right chart, the pan-genome development of the *C. pseudotuberculosis* biovar *equi* strains.

To achieve a global view of the genome repertoire of *C. pseudotuberculosis*, the pan-genome (i.e., the total number of non-redundant genes) was calculated using the abovementioned SRV method with the software EDGAR (Figure 3). The resulting pan-genome of *C. pseudotuberculosis* contained a total of 2,782 genes, which is 1.3-fold the average total number of genes in each of the 15 strains (2,078). However, when the pan-genomes of the biovars were calculated separately, a slightly different scenario emerged, in which the biovar *ovis* had a pan-genome of 2,403 genes, 1.14-fold the average total number of genes in each biovar *ovis* strain (2,098), and the biovar *equi* had a pan-genome with 2,521 genes, 1.23-fold the average total number of genes in each biovar *equi* strain (2,047).

Additionally, the extrapolation of the *C. pseudotuberculosis* pan-genome was calculated by curve fitting based on Heaps’ Law, as represented by the formula n = κ * N^−α^, where *n* is the expected number of genes for a given number of genomes, *N* is the number of genomes, and the other terms are constants defined to fit the specific curve [67]. The variables *κ* and *γ* were determined to be 2,043.06 and 0.11, respectively, by using the statistical computing language R. According to Heaps’ Law,1) an *α≤1* is representative of an open pan-genome, meaning that each added genome will contribute some new genes and the pan-genome will increase, and 2) an *α>1* represents a closed pan-genome, in which the addition of new genomes will not significantly affect the pan-genome. Using the formula *α = 1−γ*, we inferred that the pan-genome of *C. pseudotuberculosis* is increasing with an *α* of 0.89, indicating that it has an open pan-genome. The extrapolation of the pan-genome was also separately calculated for both biovars, *ovis* and *equi*. Although the biovar *equi* had the same *α* as the entire pan-genome (0.89), the biovar *ovis* had a much-higher *α* of 0.94.

### Core Genome of the Species *C. pseudotuberculosis*

**Figure 4:**
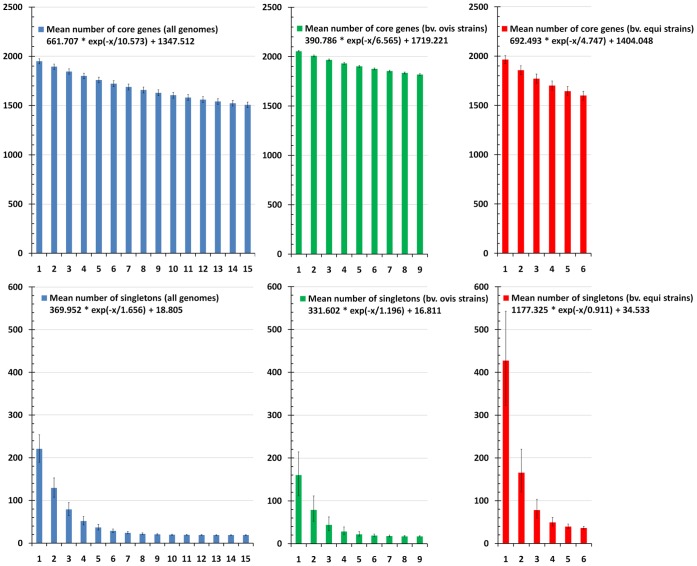
Core genome and singleton development of *C. pseudotuberculosis*. Upper-left, the core genome development using permutations of all 15 strains of *C. pseudotuberculosis*; upper-center, the core genome development of the *C. pseudotuberculosis* biovar *ovis* strains; upper-right, the core genome development of the *C. pseudotuberculosis* biovar *equi* strains; lower-left, the singleton development using permutations of all 15 strains of *C. pseudotuberculosis*; lower-center, the singleton development of the *C. pseudotuberculosis* biovar *ovis* strains; lower-right, the singleton development of the *C. pseudotuberculosis* biovar *equi* strains.

The core genome of a species is defined as the subset of genes from the pan-genome that are shared by all strains. Here, the core genome of *C. pseudotuberculosis* was calculated with the software EDGAR by defining the subset of genes that presented orthologs in all the strains using the SRV method. The subset of core genes of *C. pseudotuberculosis* contained 1,504 genes, which represented 54% of the entire pan-genome of the species (2,782 genes). This subset may decrease with the addition of new genomes, as shown by the tendency of the core genes in the blue curve (Figure 4). However, although this subset may slightly decrease, the extrapolation of the curve can be calculated by the least-squares fit of the exponential regression decay to the mean values, as represented by the formula n = κ * exp[−x/τ]+tg(θ), where *n* is the expected subset of genes for a given number of genomes, *x* is the number of genomes, *exp* is Euler’s number, and the other terms are constants defined to fit the specific curve. Interestingly, that formula can be used to predict that with a high number of genomes (*x*), the κ * exp[−x/τ] term will tend toward 0, where *tg(θ)* represents the convergence of the genome subset. Based on this observation, the core genome of *C. pseudotuberculosis* tended to converge to 1,347 genes, which represented 48% of the pan-genome of the species (2,782 genes).

**Figure 5:**
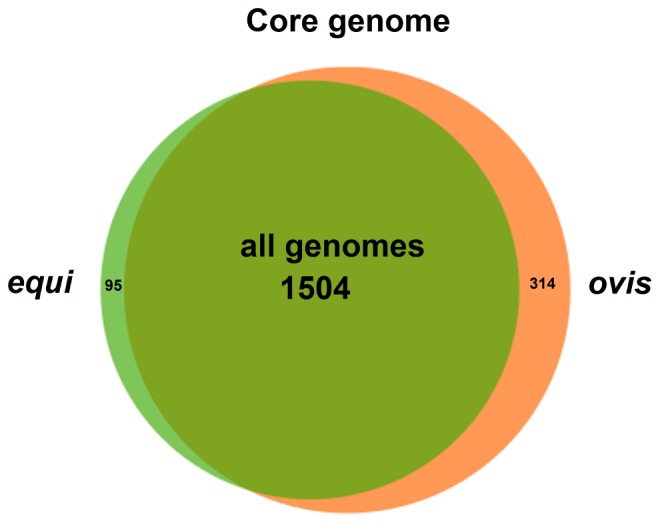
Venn diagram representing the core genomes of the *C. pseudotuberculosis* strains. All genomes, the number of genes composing the core genome of all the strains; *equi*, the number of genes of the core genome of the *C. pseudotuberculosis* biovar *equi* strains, which were absent in one or more of the *C. pseudotuberculosis* biovar *ovis* strains; *ovis*, the number of genes of the core genome of the *C. pseudotuberculosis* biovar *ovis* strains, which were absent in one or more of the *C. pseudotuberculosis* biovar *equi* strains.

The separate analyses of the core genomes of biovars *ovis* and *equi* (Figure 4) presented different scenarios. The core genome of the *C. pseudotuberculosis* biovar *ovis* strains contained 1,818 genes, and it tended to stabilize at approximately 1,719 genes, according to the exponential regression decay. The *C. pseudotuberculosis* biovar *equi* strains, however, presented a more compact core genome of 1,599 genes and tended to stabilize at 1,404 genes. Altogether, with a total *C. pseudotuberculosis* core genome of 1,504 genes and a biovar *ovis* core genome of 1,818 genes, the core genome of biovar *ovis* is predicted to contain 314 orthologous genes that are shared by all strains from this biovar and are absent from one or more strains of biovar *equi* (Figure 5). Additionally, using the same strategy, the biovar *equi*, with 1,599 genes, contained 95 core genes that were absent from one or more strains of biovar *ovis* (Figure 5).

**Figure 6:**
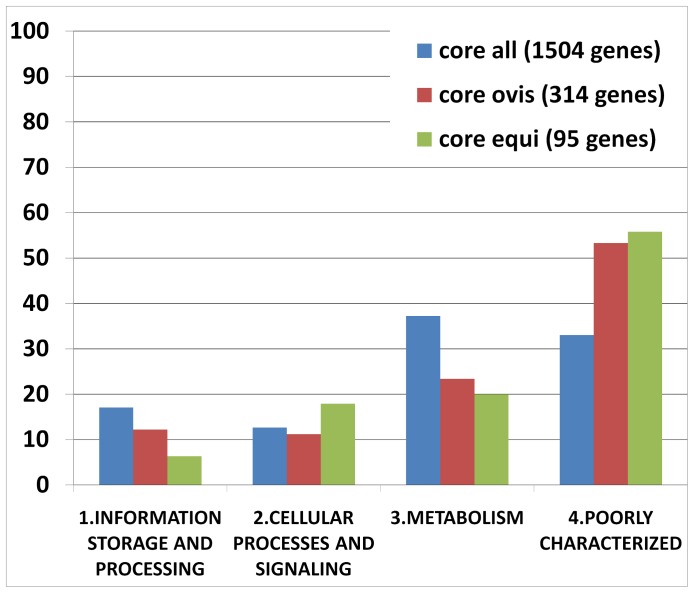
Core genes of the *C. pseudotuberculosis* strains classified by COG functional category. Core all, the genes composing the core genome of all the strains; core *ovis*, the genes of the core genome of the *C. pseudotuberculosis* biovar *ovis* strains, which were absent in one or more of the *C. pseudotuberculosis* biovar *equi* strains; core *equi*, the genes of the core genome of the *C. pseudotuberculosis* biovar *equi* strains, which were absent in one or more of the *C. pseudotuberculosis* biovar *ovis* strains.

The core genome of all the strains and the differential core genome of the biovar *ovis* and *equi* strains were classified by COG functional category. According to the chart in Figure 6, the core genome of all the strains had a large number of genes related to the categories “Metabolism” and “Information storage and processing”. Moreover, a high proportion of the core genome of all the strains was classified as “Poorly characterized”. However, when analyzing the differential core genes of the biovar *ovis* and *equi* strains separately, a higher proportion of “Poorly characterized” genes was clearly detected in the differential core genes when compared with the core genome of all the strains (Figure 6). Finally, the biovar *equi* had a larger number of genes classified under the functional category “Cellular processes and signaling” than biovar *ovis* strains.

### Singletons: Strain-specific Genes Detected in the Species *C. pseudotuberculosis*

The singletons of a strain are defined as the subset of genes that are absent from all the other strains and are thus responsible for increases in the number of genes in the pan-genome. We used the SRV method and EDGAR to calculate the subset of *C. pseudotuberculosis* singletons as the genes that did not present orthologs in any other strain. Moreover, by the least-squares fit of the exponential regression decay to the mean values, as previously described by the formula n = κ * exp[−x/τ]+tg(θ), we calculated the *tg(θ)* (Figure 4) for the three datasets: A) all the genomes, B) the biovar *ovis* genomes, and C) the biovar *equi* genomes. The *tg(θ)* for all the genomes was 18.805, meaning that each sequenced genome added approximately 19 genes to the total gene pool of the species *C. pseudotuberculosis*, i.e., the pan-genome. However, the individual analysis of each biovar revealed a scenario in which each sequenced biovar *ovis* strain contributed ∼16 genes, but each sequenced biovar *equi* strain contributed ∼34 genes.

### Detection of PAIs in the *C. pseudotuberculosis* Genomes

Intraspecies genome plasticity may result from several events, of which horizontal gene transfer is particularly important because it can cause the acquisition of blocks of genes (genomic islands, or GEIs), producing evolution by quantum leaps [76]. PAIs are important in this context because they represent a class of GEIs that carry virulence genes, i.e., factors that enable or enhance the parasitic growth of an organism inside a host [77]. Therefore, high concentrations of the two following subsets of genes would be expected inside PAIs: 1) shared genes, which are shared by two or more, but not all, strains; and 2) singletons.

In previous studies, seven PAIs were identified in *C. pseudotuberculosis* biovar *ovis* strains 1002 and C231 (PiCps 1–7) [43], and four additional PAIs have been identified in *C. pseudotuberculosis* strain 1002 by further comparisons with *C. pseudotuberculosis* strains 316 and 258 (PiCps 8–11) [54]–[56]. The latter subset of PAIs was identified due to a better view of the two biovars and their specific patterns of plasticity. Here, we applied the same methodology used in the previous studies, using the software PIPS to achieve a global view of the PAIs in 15 *C. pseudotuberculosis* strains. Briefly, in addition to the previously identified 11 PAIs, we found 5 new PAIs, identified as PiCps 12–16. Although the 16 PAIs are present in all strains, they have different patterns of deletions, especially in the biovar *equi* strains (Figure 2). PiCp1, as previously described [43], harbors the *pld* gene and the *fag* operon and is present in all the strains. PiCp3 harbors the diphtheria toxin gene (*tox*) in *C. pseudotuberculosis* strain 31, and PiCps 7 and 15 harbor the *spaD* and *spaA* pilus gene clusters, respectively.

**Figure 7:**
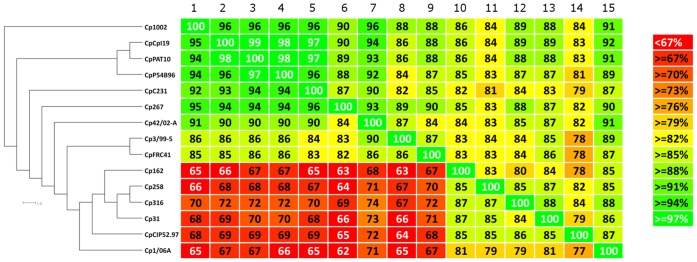
Phylogenomic tree and heatmap analyses of the *Corynebacterium pseudotuberculosis* strains based on pathogenicity island plasticity. Comparisons between the PAI contents of all the strains were plotted as percentages of similarity on the heatmap using Gegenees (version 1.1.4). The percentages of similarity were used to generate a phylogenomic tree with SplitsTree (version 4.12.6). Numbers from 1 to 15 (upper-left to upper-right corner) represent the strains from Cp1002 to Cp1/06-A (upper-left to lower-left corner). On the heatmap, the upper portion is not symmetrical to the lower portion because the pathogenicity islands contents of all genomes present different sizes. Therefore, considering a scenario where the pathogenicity islands content from genomes A and B are composed of 100 and 80 genes, respectively, with a common repertoire of 40 genes, genome A will present 40% of similarity to genome B and genome B will present 50% of similarity to genome A.

To assess the level of plasticity in the PAIs, we used the orthologous data predicted by EDGAR to calculate the percentage of PAIs (from each strain) present in each of the other strains. Using these data, we generated a phylogenomic tree of the strains with SplitsTree (Figure 7). The phylogenomic tree produced a clear separation of the *ovis* and *equi* biovar strains, similar to the phylogenomic tree created using Gegenees (Figure 1). A further comparison of the Gegenees and PAI phylogenomic trees revealed that the latter strategy did not cluster *C. pseudotuberculosis* strains 42/02-A and C231 in the same branch as did the former. However, two other branches were in agreement with the phylogenomic tree created by Gegenees: *C. pseudotuberculosis* strains 258 and 316 clustered together in a biovar *equi* group, and *C. pseudotuberculosis* strains 3/99-5 and FRC41 clustered in a biovar *ovis* group.

Additionally, we used the comparison data generated by the PAI analyses to create a new heatmap (Figure 7), from which we deduced a high level of intra-biovar similarity in the *ovis* strains with respect to the PAI content (82–100%). Although biovar *ovis* showed a lower level of similarity to biovar *equi* with respect to the PAI content (78–91%), the former tended to present a similar deletion pattern in the same PAIs, independent of the strain. The biovar *equi* strains, however, contained large deletions and a lower level of similarity intra-biovar (77–88%) and also compared with the biovar *ovis* PAIs (62–74%) (Figure 2A).

### Variations in Pathogenicity Islands Encoding Exotoxin Virulence Factors

As described previously, the major toxin of *C. pseudotuberculosis* is phospholipase D (PLD), which is encoded by the *pld* gene and is strongly associated with the spread of bacteria throughout the host cells [1]. In a previous study, this toxin was shown to be harbored by a PAI (PiCp1) close to the *fag* operon, which also encodes important virulence factors that are responsible for iron acquisition in environments where this element is scarce [43]. Here, we found that the *pld* gene was present in 14 of 15 strains, with similarities ranging from 98–100%. This finding was expected due to the important role of PLD during the disease course; *pld* mutants present a diminished ability to spread throughout the host [1].

Although the *pld* gene plays a pivotal role in pathogenesis, *C. pseudotuberculosis* strain 31 contains a frameshift mutation near the 3′-end of this gene that could decrease the ability of this strain to spread throughout the host. However, *C. pseudotuberculosis* strain 31 was the only strain in our dataset to present another important virulence factor, the diphtheria toxin gene (*tox*) (Cp31_0135). The diphtheria toxin (DT) is an important virulence factor in *C. diphtheriae*, in which the gene was acquired through lysogenization by corynephages, meaning that the *tox* gene is also present in a PAI in this species and can be horizontally transferred to other organisms. Briefly, the *tox* gene is regulated by the chromosomal iron-dependent repressor DtxR [78], which blocks the transcription process by binding to the *tox* operator [79]. When gene transcription is activated, the toxin precursor is exported and cleaved into two fragments (A and B), which are joined by a disulfide bond [80]; fragment B binds the membrane of the host cell, mediating the internalization of fragment A, which exhibits ADP-ribosyltransferase activity [79], [81].

The exotoxin catalizes the transfer of adenosine diphosphate ribose (ADP-ribosylation) from nicotinamide adenine dinucleotide (NAD) to a histidine residue of elongation factor 2 (EF-2), called diphthamide. This process leads to inactivation of EF-2 and inhibits chain elongation during protein synthesis [82]. This toxin has also been identified in *C. ulcerans* strains, where it causes diphtheria-like illness [83], [84], and, interestingly, in two *C. pseudotuberculosis* strains isolated from buffalo in Egypt [10], [85]. The *tox* gene from *C. pseudotuberculosis* 31 has 560 amino acids in length, does not present any frameshift and has ∼96–97% similarity to the *tox* genes from several *C. diphtheriae* strains and from corynephage β, as well as ∼94–95% similarity to the *tox* gene from *C. ulcerans* 0102 (data not shown). Given the absence of the *pld* gene, the similarity of the *tox* gene from *C. pseudotuberculosis* to those from the *C. diphtheriae* strains, the conservation of all the domains and the presence of the gene in other strains isolated from buffalo in Egypt, the following question can be raised: is DT required for *C. pseudotuberculosi*s to infect buffalo or is this feature more closely related to the geographical location (Egypt) than to the host?

### Variations and Deletions Detected in PiCps 4, 5 and 9

Specific patterns of deletions in PiCps 4, 5 and 9 of *C. pseudotuberculosis* CIP52.97, 316 and 258 (biovar *equi* strains) have been demonstrated [54]–[56]. Here, we detected the same deletions in all the biovar *equi* strains, which indicates that these deletion events were specific to the mentioned biovar (Figure S1). Although most of the deleted CDSs encoded hypothetical or phage proteins (integrases and phage-associated proteins), one gene of PiCp5 encoded a putative sigma 70 factor (Cp1002_1452) and deserves attention because it is most likely involved in the correct assembly of the transcription machinery at specific promoters and is therefore associated with the general transcription process [43].

### Differences between Pilus Gene Clusters Located on PiCp15 and PiCp7

According to work performed by Yanagawa and Honda in 1976 [47], *C. pseudotuberculosis* cells possess pilus structures, although the number of pili per bacterial cell is small, and at times, a long bundle measuring more than several micrometers in length was the only pilus observed. In a more recent genomic study, two clusters of pilus genes were described in *C. pseudotuberculosis* FRC41 and were named according to their major pilin gene: the *spaA* (*srtB*-*spaA*-*srtA*-*spaB*-*spaX*-*spaC*) and *spaD* (*srtC*-*spaD*-*spaY*-*spaE*-*spaF*) clusters, where *srtA* and *srtB* are the specific sortases of the *spaA* cluster; *spaA*, *spaB* and *spaC* encode the major, base and tip pilin proteins, respectively, of the *spaA* cluster; *srtC* is the specific sortase of the *spaD* cluster; *spaD*, *spaE* and *spaF* encode the major, base and tip pilin proteins, respectively, of the *spaD* cluster; and *spaX* and *spaY* have currently unknown functions. Additionally, a housekeeping sortase (*srtD*) is likely responsible for anchoring the pili to the cellwall [46].

**Figure 8:**
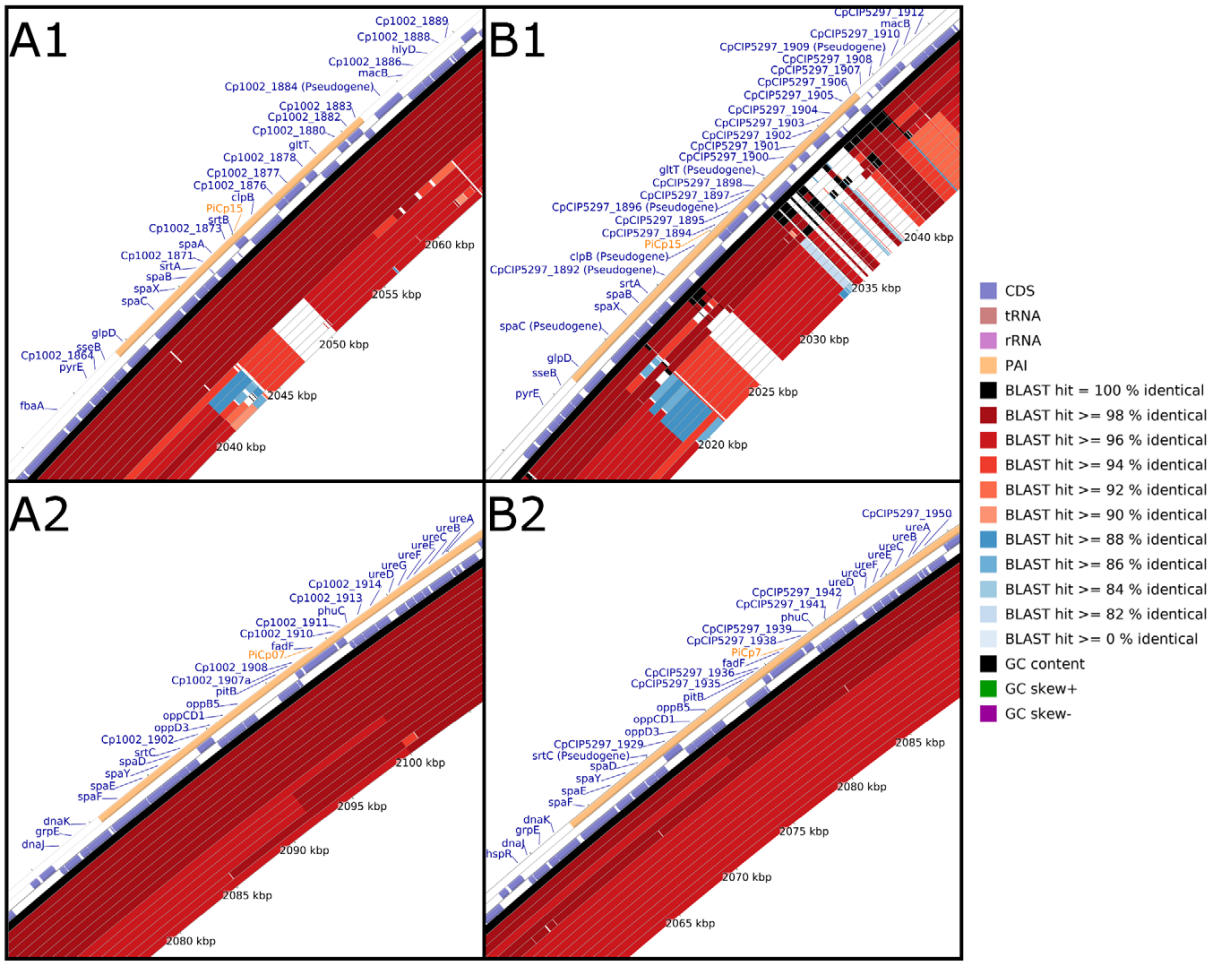
Plasticity of the pilus gene clusters *spaA* and *spaD* in *C. pseudotuberculosis*. A1 and B1, PiCp15 harboring the *spaA* cluster of genes; A2 and B2, PiCp7 harboring the *spaD* cluster of genes. A, all the *C. pseudotuberculosis* strains were aligned using *C. pseudotuberculosis* strain 1002 as a reference. From the inner to outer circle on A1 and A2: the biovar *equi* strains Cp31, Cp1/06-A, CpCp162, Cp258, Cp316, CpCIP52.97; and, the biovar *ovis* strains CpC231, CpP54B96, Cp267, CpPAT10, CpI19, Cp42/02-A, Cp3/99-5, CpFRC41 and Cp1002. B, all the *C. pseudotuberculosis* strains were aligned using *C. pseudotuberculosis* strain CIP52.97 as a reference. From the inner to outer circle on B1 and B2: the biovar *ovis* strains CpC231, Cp1002, CpPAT10, Cp267, CpP54B96, CpI19, Cp42/02-A, CpFRC41, Cp3/99-5, Cp1/06-A; and, the biovar *equi* strains Cp31, CpCp162, Cp316, Cp258 and CpCIP52.97. CDS, coding sequences; tRNA, transfer RNA; rRNA, ribosomal RNA; and PAI, pathogenicity island.

Interestingly, the *spaA* and *spaD* gene clusters were located in PAIs (PiCps 15 and 7, respectively) (Figure 8), which is in agreement with the presence of pilin genes in horizontally acquired regions of Gram-negative and Gram-positive bacteria, such as *Vibrio cholerae* and *C. diphtheriae*, respectively [86], [87]. Moreover, although the biovar *ovis* strains had a complete *spaA* cluster, the biovar *equi* strains contained a large deletion at the position where the *spaA* and *srtB* genes should be located (PiCp15). Furthermore, the entire *srtA*-*spaB*-*spaX*-*spaC* region presented a low similarity to the same region in the biovar *ovis* strains, which was caused by small deletions, frameshift mutations and nucleotide substitutions (Figure 8).

With respect to the *spaD* cluster of the biovar *ovis* strains, the major pilin gene *spaD* contains a frameshift in *C. pseudotuberculosis* P54B96 and PAT10; and in *C. pseudotuberculosis* 267, the tip pilin gene *spaF* also contains a frameshift. In biovar *equi* strains, the *spaD* gene of all the strains had 99% similarity to the *spaD* gene of the biovar *ovis* strains. However, *C. pseudotuberculosis* CIP52.97 contains a frameshift mutation in the specific sortase gene *srtC*. Furthermore, the base and tip pilin genes, *spaE* and *spaF*, respectively, of *C. pseudotuberculosis* strains 258, 316, 1/06-A and Cp162 are merged into the same reading frame.

## Discussion

### *Corynebacterium pseudotuberculosis* – all Strains

According to the *rpoB* gene tree generated by Khamis *et al.* [88], *C. jeikeium*, *C. urealyticum*, *C. kroppenstedtii* and *C. variabile* cluster together in group 3, and *C. aurimucosum* appears in group 1. Moreover, *C. glutamicum* and *C. efficiens* cluster together in one branch, whereas *C. pseudotuberculosis*, *C. diphtheriae* and *C. ulcerans* appear closely related in another branch. Furthermore, *C. ulcerans* appears closer to *C. pseudotuberculosis* than to *C. diphtheriae*. Based on our results, we can deduce that although many variable regions exist between the pathogenic members of the genus *Corynebacterium*, these species tend to cluster together because they most likely share some core virulence determinants. Finally, although *C. kroppenstedtii* did not cluster with group 3, the other species were in perfect agreement with the *rpoB* analysis of Khamis *et al.* [88].

Two striking characteristics of *C. kroppenstedtii* are the absence of mycolic acids in the cell wall (due to the losses of a condensase gene cluster and a mycolate reductase gene) and a lipophilic phenotype (due to the absence of a microbial type I fatty acid synthase gene) [89]. Therefore, the transitional phylogenomic position of *C. kroppenstedtii* between the pathogenic and non-pathogenic species was in agreement with the lack of important virulence genes and the low pathogenic potential characteristic of *C. kroppenstedtii* [89]–[91].

At the species level, the heatmap indicated a clonal-like behavior of *C. pseudotuberculosis* compared with the *C. diphtheriae* species. Trost *et al.* [87] have highlighted the high plasticity of the *C. diphtheriae* genome, which is mainly related to the 57 genomic islands identified in this species. With respect to the clonal-like behavior of *C. pseudotuberculosis*, Bolt [92] have identified 10 STs among 73 strains of *C. pseudotuberculosis* typed by MLST, where 7 and 4 STs were associated with 64 and 9 strains of biovar *ovis* and *equi*, respectively. The few number of STs identified by MLST was in agreement with previous typing studies [17], [93], [94] in that the strains of *C. pseudotuberculosis* are clonally related. Moreover, although there were 7 STs identified for biovar *ovis* strains, 6 and 7 of them were clustered in one sole eBURST group when considering single locus variants (SLVs) and double locus variants (DLVs), respectively; and, all the STs identified for biovar *equi* shared two alleles with the biovar *ovis* strains [92]. Finally, the MLST findings indicate that: 1) biovar *ovis* and *equi* strains share a common evolutionary origin, although they are now relatively distinct genotypic clusters; and, 2) biovar *ovis* is a clonal-like organism. Our results with respect to this clonal-like behavior of *C. pseudotuberculosis* are also in agreement with PFGE data from Connor *et al.* [17] and can also be inferred from the extrapolation of the pan-genome data, in which *C. pseudotuberculosis* had a slightly higher *α* value of 0.89 compared with the *C. diphtheriae α* value of 0.69; and, from the total number of genes in the pan-genome of *C. pseudotuberculosis* (2,782 genes), which is compact compared with that of the closely related species *C. diphtheriae*, which contains 4,786 genes [87].

Although *C. pseudotuberculosis* displays some clonal-like behavior, the resulting *α* of 0.89 from the extrapolation of the pan-genome indicates that it has an open pan-genome. Moreover, considering that *α* is inversely proportional to the pan-genome increasing rate, in contrast to the *C. diphtheriae α* of 0.69, the *α* of 0.89 of the *C. pseudotuberculosis* pan-genome indicates that the latter is increasing at a slower rate. This slow increase is related to the low number of singletons (∼19) added to the pan-genome of *C. pseudotuberculosis* by each newly sequenced strain, whereas each strain of *C. diphtheriae* added ∼65 genes to the entire pan-genome [87]. Moreover, the slow increase and higher *α* value are in agreement with the intracellular facultative behavior of this species. Because strictly intracellular organisms tend to have closed pan-genomes due to their limited contact with potential gene donors, an intracellular facultative organism such as *C. pseudotuberculosis*, even when it has different hosts, can be expected to have an *α* that is closer to 1 than that of *C. diphtheriae* [95], [96].

With respect to the core genome of all the strains, a large number of genes are related to the categories “Metabolism” and “Information storage and processing”. The “Information storage and processing” category contains genes involved in translation, ribosomal structure and biogenesis, RNA processing and modification, transcription, replication, recombination and repair, and other important functions; the “Metabolism” category contains genes involved in the production and conversion of energy, as well as the transport and metabolism of carbohydrates, amino acids, nucleotides, coenzymes, lipids, inorganic ions and secondary metabolites. Given the importance of the core genome, these two functional categories are expected to be highly represented in the analyzed subset. Finally, although a large number of “Poorly characterized” genes were identified in the core gene subset, this result is in agreement with previous core genome analyses of *Aggregatibacter actinomycetemcomitans*, in which one-third of the genes were categorized as “Poorly characterized” and approximately one-third were classified under “Metabolism” [97].

### *Corynebacterium pseudotuberculosis* – Biovars *Ovis* and *Equi*

Connor *et al.* [17] and Bolt [92] have investigated the clonal aspect of *C. pseudotuberculosis* using PFGE and MLST, respectively, which enabled them to differentiate the *equi* and *ovis* biovars. On the phylogenomic tree, the *C. pseudotuberculosis* genomes also clustered in two separate groups representing the two biovars of the species: biovar *ovis*, with more than 99% similarity according to the heatmap; and biovar *equi*, with a similarity ranging from 95% to almost 100%. This result highlights the higher plasticity of *C. pseudotuberculosis* biovar *equi* compared with the biovar *ovis* strains, although this plasticity is not as high as that described for *C. diphtheriae* strains. Moreover, the same conclusion (regarding the relative plasticity of the two biovars) may be drawn from the number of singletons, in which the biovar *equi* strains presented higher levels of variability in the number of singletons, compared with the biovar *ovis* strains (Table 1). The circular genome comparison generated by CCT also revealed the clonal-like behavior of biovar *ovis*, with all the *ovis* strains containing minor deletions compared with *C. pseudotuberculosis* strain 1002 (Figure 2A); and the presence of a higher number of singletons in biovar *equi*, with all the strains from both biovars presenting similar deletion patterns when compared with *C. pseudotuberculosis* strain CIP52.97 (Figure 2B). Finally, the majority of the genomic variations on the circular genome comparison were found in PAI regions, which are very important for virulence potential and host adaptation and are known as mosaic and unstable [69].

Interestingly, the analysis of the pan-genome subsets revealed that the *ovis* and *equi* biovar strains contain major variations of the data found in the entire pan-genome. Although the pan-genome of biovar *equi* had an invariable *α* value of 0.89, the pan-genome of the biovar *ovis* had a higher *α* value of 0.94, which was strictly correlated to the higher clonal-like behavior of this biovar compared with biovar *equi* [92]. Moreover, its high *α* value and the pan-genome curve suggest that the pan-genome of biovar *ovis* is increasing at a slower rate than that of biovar *equi*.

The same conclusion may be drawn from the development of singletons: each biovar *ovis* strain added ∼16 singletons to the pan-genome, but each biovar *equi* strain added ∼34 singletons to the gene pool. Moreover, although the core genome subset of the biovar *ovis* strains (1,818 CDS) was slightly higher than that of the biovar *equi* strains (1,599 CDS), most of the variable genes of the biovar *ovis* strains were acquired in blocks through horizontal gene transfer and are highly conserved throughout the entire biovar, as shown in Figure 2A. In contrast, the biovar *equi* strains presented great variability, both intra- and inter-biovar, in the content of the detected pathogenicity islands (Figure 2B). Finally, a comparison of the similarity levels on the two heatmaps, generated by Gegenees (93–100%, Figure 1) and from PAI contents (62–100%, Figure 7), also revealed that most of the variability defining the biovars *ovis* and *equi* arose from the gene content of the PAIs.

In view of this, one possible explanation for the large number of “Poorly characterized” genes in the differential core subsets of both biovars *ovis* and *equi* is the abovementioned acquisition of these subsets by horizontal gene transfer, which tends to involve a large number of hypothetical proteins [98], and the maintenance of these acquired regions in different biovars because they enabled the biovars to colonize specific hosts. Finally, the higher proportion of the functional category “Cellular processes and signaling” in biovar *equi* is most likely related to host adaptation because many genes in this cluster had functions such as defense mechanisms, signal transduction mechanisms, cell wall/membrane/envelope biogenesis, cell motility, and extracellular structures.

### Variations in Pilus Gene Clusters

With respect to the gene content of the PAIs, the most interesting finding is the high similarity of the pilus genes in the biovar *ovis* strains, which is in contrast to the large variability of these genes in the biovar *equi* strains. Pilus gene clusters are normally acquired in a block through horizontal gene transfer and are composed of a specific sortase gene and the major, base and tip pilin genes. Briefly, the specific sortase protein of each cluster is responsible for cleaving the LPxTG motif of the major, base and tip pilin proteins of that cluster between the threonine (T) and glycine (G) amino acids, capturing the cleaved polypeptides, polymerizing the monomers, and transferring the final product to the housekeeping sortase of the bacterium for its final incorporation into the cell wall [99], [100]. In the absence of a housekeeping sortase, the pilus-specific sortase can mediate the incorporation of the polymer into the cell wall. However, the presence of both housekeeping and specific sortases is necessary to efficiently anchor the pilus to the cell wall [101]. Moreover, although the expression of the major pilin is absolutely required for the specific pilus polymerization, the base and tip pilin monomers may still attach to the cell wall in its absence [100]–[103].

Although the biovar *ovis* strains present a complete *spaA* cluster, the biovar *equi* were shown to present large deletions in this cluster. Because of the deletion of the major pilin SpaA in the biovar *equi*, the base and tip pilin monomers would be expected to be the only pilin structures that could attach to the cell wall in a non-polymerized manner. Moreover, the deletion of one of the specific sortase genes in biovar *equi*, *srtB*, could also interfere in the efficient cell wall-anchoring of these monomers, causing them to be secreted [101]. Finally, even the production and sizes of these proteins may vary among the biovar *equi* strains because these proteins contain small deletions and frameshift mutations. Altogether, the differences in the *spaA* cluster of the biovar *equi* strains could account for the different levels of host cell attachment compared with the biovar *ovis* strains and even among the biovar *equi* strains, as found in the *C. diphtheriae* species 87,104,105.

In contrast to the high similarity found between the *spaA* clusters of the biovar *ovis* strains, the *spaD* clusters presented differences in three strains of this biovar. In *C. pseudotuberculosis* P54B96 and PAT10, a frameshit in the major pilin gene *spaD* impairs the coding of the entire protein and, thus, the polymerization of the pilin structure; and, in *C. pseudotuberculosis* 267, the tip pilin gene *spaF* also contains a frameshift. Although the tip pilin is not required for the polymerization of the pilin structure and adhesion to the host cell wall, its absence can slightly decrease the degree of adherence, which could reduce the spread of *C. pseudotuberculosis* strain 267 [106]. With respect to the *spaD* cluster of the biovar *equi* strains, a frameshift mutation in the specific sortase gene *srtC* of *C. pseudotuberculosis* CIP52.97 prevents the polymerization of the pilin structure. Moreover, the base and tip pilin genes, *spaE* and *spaF*, respectively, of *C. pseudotuberculosis* strains 258, 316, 1/06-A and Cp162 are merged into the same reading frame. Overall, these results suggest that although *C. pseudotuberculosis* 258, 316, 1/06-A and Cp162 can polymerize the major pilin, *C. pseudotuberculosis* strain 31 is most likely the only biovar *equi* strain able to polymerize an entire pilin structure from the *spaD* cluster, whereas all the biovar *ovis* strains are likely capable of producing one or two types of pilin structures (*spaA* and *spaD*).

Summarizing, all the *C. pseudotuberculosis* biovar *ovis* strains likely contain a functional *spaA* cluster of pilus genes; only three strains (267, P54B96 and PAT10) are unable to polymerize an entire *spaD* pilin structure (most likely, they instead attach monomers or incompletely polymerized pilin structures). In contrast, all the biovar *equi* strains contain deletions, which render them unable to polymerize any *spaA* pilin structures; within this biovar, only *C. pseudotuberculosis* 31 appears to be able to polymerize an entire *spaD* pilin structure. Given the pivotal role played by pili in the processes of adhesion and internalization, the polymerization of complete pilin structures in the biovar *ovis* strains could be responsible for the great ability of these strains to spread throughout host tissues and penetrate cells to grow intracellularly [48], [101], [106], [107]. Based on this observation, the biovar *ovis* strains are expected to have less contact with other organisms than the biovar *equi* strains and to therefore show more clonal-like behavior. Finally, these results could also explain the distinct pattern of the diseases caused by *C. pseudotuberculosis* in horses, which involves ulcerative lymphangitis that rarely evolves to a visceral form [108]. However, more studies are needed to assess whether the *C. pseudotuberculosis* biovars *equi* and *ovis* truly present different patterns of pilin formation and, thus, variable degrees of host tissue adhesion, spreading and cell internalization.

## Supporting Information

Figure S1 Plasticity of PiCps 4, 5 and 9. A1 and B1, PiCp9; A2 and B2, PiCp4; A3 and B3, PiCp5. A, all the *C. pseudotuberculosis* strains were aligned using *C. pseudotuberculosis* strain 1002 as a reference. From the inner to outer circle on A1, A2 and A3: the biovar *equi* strains Cp31, Cp1/06-A, CpCp162, Cp258, Cp316, CpCIP52.97; and, the biovar *ovis* strains CpC231, CpP54B96, Cp267, CpPAT10, CpI19, Cp42/02-A, Cp3/99-5, CpFRC41 and Cp1002. B, all the *C. pseudotuberculosis* strains were aligned using *C. pseudotuberculosis* strain CIP52.97 as a reference. From the inner to outer circle on B1, B2 and B3: the biovar *ovis* strains CpC231, Cp1002, CpPAT10, Cp267, CpP54B96, CpI19, Cp42/02-A, CpFRC41, Cp3/99-5, Cp1/06-A; and, the biovar *equi* strains Cp31, CpCp162, Cp316, Cp258 and CpCIP52.97. CDS, coding sequences; tRNA, transfer RNA; rRNA, ribosomal RNA; and PAI, pathogenicity island.
(TIFF)
