# Supplementary figures and images for: The Pan-Genome of the Animal Pathogen Corynebacterium pseudotuberculosis Reveals Differences in Genome Plasticity between the Biovar ovis and equi Strains
Source: PLoS One. 2013 Jan 14;8(1):e53818. doi: 10.1371/journal.pone.0053818 (PMC3544762; doi:10.1371/journal.pone.0053818)

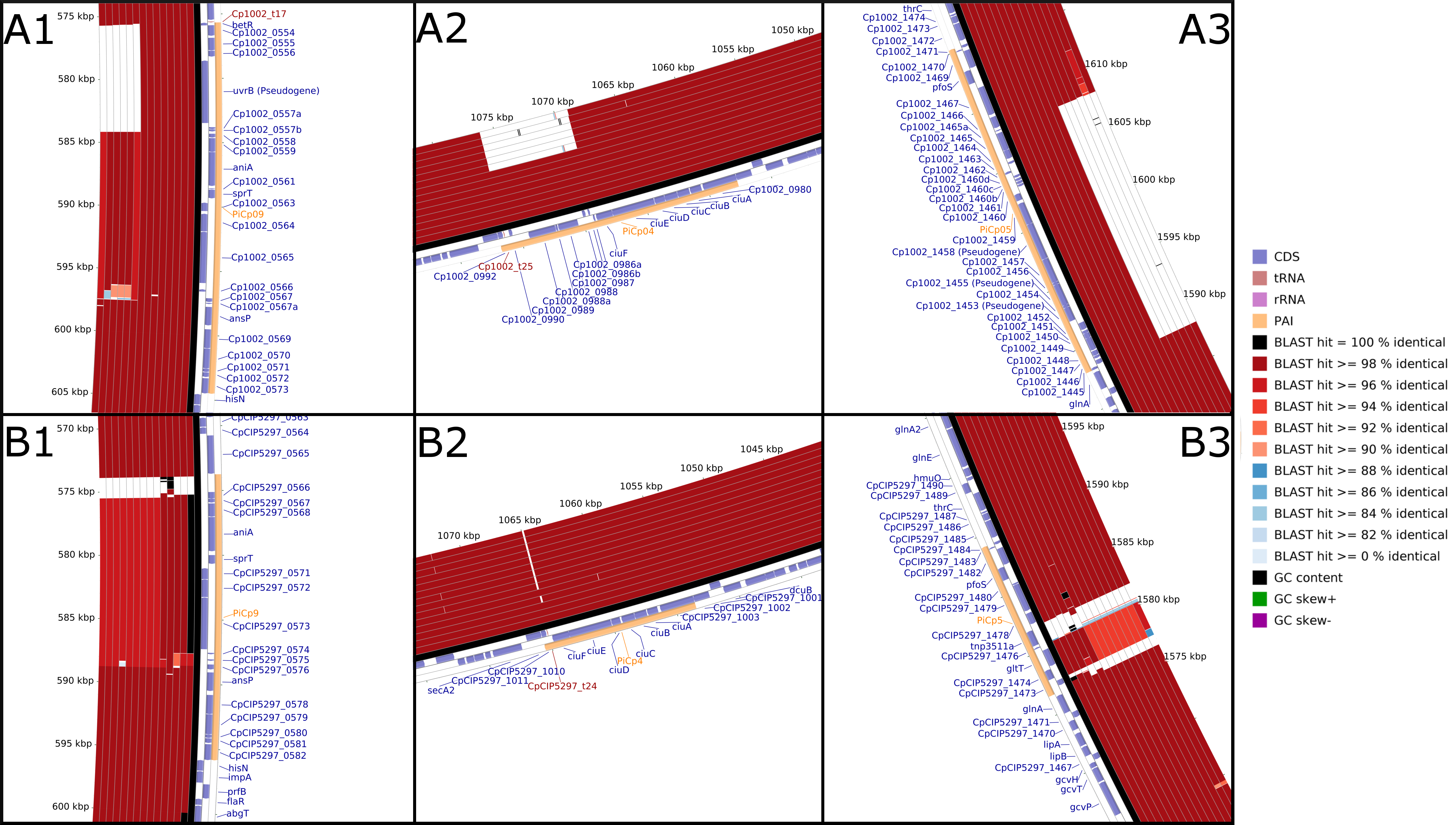

Supplement: Figure S1 — Plasticity of PiCps 4, 5 and 9. A1 and B1, PiCp9; A2 and B2, PiCp4; A3 and B3, PiCp5. A, all the C. pseudotuberculosis strains were aligned using C. pseudotuberculosis strain 1002 as a reference. From the inner to outer circle on A1, A2 and A3: the biovar equi strains Cp31, Cp1/06-A, CpCp162, Cp258, Cp316, CpCIP52.97; and, the biovar ovis strains CpC231, CpP54B96, Cp267, CpPAT10, CpI19, Cp42/02-A, Cp3/99-5, CpFRC41 and Cp1002. B, all the C. pseudotuberculosis strains were aligned using C. pseudotuberculosis strain CIP52.97 as a reference. From the inner to outer circle on B1, B2 and B3: the biovar ovis strains CpC231, Cp1002, CpPAT10, Cp267, CpP54B96, CpI19, Cp42/02-A, CpFRC41, Cp3/99-5, Cp1/06-A; and, the biovar equi strains Cp31, CpCp162, Cp316, Cp258 and CpCIP52.97. CDS, coding sequences; tRNA, transfer RNA; rRNA, ribosomal RNA; and PAI, pathogenicity island. (TIFF) [file pone.0053818.s001.tiff]
